# Comparative Study on Gluten Protein Composition of Ancient (Einkorn, Emmer and Spelt) and Modern Wheat Species (Durum and Common Wheat)

**DOI:** 10.3390/foods8090409

**Published:** 2019-09-12

**Authors:** Sabrina Geisslitz, C. Friedrich H. Longin, Katharina A. Scherf, Peter Koehler

**Affiliations:** 1 Leibniz-Institute for Food Systems Biology at the Technical University of Munich, Lise-Meitner-Strasse 34, 85354 Freising, Germany; 2 State Plant Breeding Institute, University of Hohenheim, Fruwirthstraße 21, 70599 Stuttgart, Germany; 3 Department of Bioactive and Functional Food Chemistry, Institute of Applied Biosciences, Karlsruhe Institute of Technology (KIT), Adenauerring 20a, 76131 Karlsruhe, Germany; 4 biotask AG, Schelztorstrasse 54-56, 73728 Esslingen am Neckar, Germany

**Keywords:** baking quality, gliadin, glutenin, Bradford assay, Osborne fractions, reversed-phase high-performance liquid chromatography (RP-HPLC)

## Abstract

The spectrophotometric Bradford assay was adapted for the analysis of gluten protein contents (gliadins and glutenins) of spelt, durum wheat, emmer and einkorn. The assay was applied to a set of 300 samples, including 15 cultivars each of common wheat, spelt, durum wheat, emmer and einkorn cultivated at four locations in Germany in the same year. The total protein content was equally influenced by location and wheat species, however, gliadin, glutenin and gluten contents were influenced more strongly by wheat species than location. Einkorn, emmer and spelt had higher protein and gluten contents than common wheat at all four locations. However, common wheat had higher glutenin contents than einkorn, emmer and spelt resulting in increasing ratios of gliadins to glutenins from common wheat (< 3.8) to spelt, emmer and einkorn (up to 12.1). With the knowledge that glutenin contents are suitable predictors for high baking volume, cultivars of einkorn, emmer and spelt with good predicted baking performance were identified. Finally, spelt, emmer and einkorn were found to have a higher nitrogen partial factor productivity than common and durum wheat making them promising crops for a more sustainable agriculture.

## 1. Introduction

The “ancient” wheats einkorn (*Triticum monococcum* L., diploid), emmer (*T. dicoccum* L., tetraploid) and spelt (*T. aestivum* ssp. *spelta*, hexaploid) have been cultivated in very low amounts compared to the “modern” wheat species common wheat (*T. aestivum* L., hexaploid) and durum wheat (*T. durum* L., tetraploid) in the 20th century. The reasons for the low cultivation of ancient wheats are 30–60% lower grain yields, the presence of husks and poor baking properties compared to common wheat [1]. Nevertheless, ancient wheats have been rediscovered in the last 20 years, because a growing number of consumers associate their consumption with sensory and health benefits due to their comparatively higher contents of e.g., ferulic acid, vitamins, alkylresorcinols and lutein [2,3,4,5,6,7,8].

Common wheat is most suitable for bread making, because the flour forms a viscoelastic dough with a high gas holding capacity when it is mixed with water. In contrast, flours of ancient wheats yield softer dough with low elasticity and high extensibility because of their poor gluten quality [1,9,10,11].

It is generally accepted that gluten proteins are one of the most important factors determining the baking quality of wheat flours. Gluten proteins are storage proteins and classified into gliadins (GLIA) soluble in aqueous alcohol and glutenins (GLUT) soluble in aqueous alcohol only after reduction of disulfide bonds. Not only the amount, but the ratio between GLIA and GLUT (GLIA/GLUT) has been shown to be responsible for good baking quality. GLIA/GLUT of common wheat is typically 1.5–3.1 [12,13], but a recent study showed that the GLIA/GLUT of ancient wheats was much higher (spelt: 2.8–4.0; emmer: 3.6–6.7; einkorn: 4.2–12.0) [11]. However, only samples grown at a single location were used in this study and it is known that the soil and climatic conditions combined with fertilization have a large impact on protein quantity and quality. We previously identified the GLUT content and GLIA/GLUT as good predictors of baking quality and, thus, high bread volume of flours from both modern and ancient wheats [11].

The contents and composition of GLIA and GLUT are typically analyzed by modified Osborne fractionation followed by reversed-phase high-performance liquid chromatography (RP-HPLC) [12]. With this technique, it is possible to analyze individual GLIA-types (α-, γ-, ω1,2- and ω5-gliadins) and high- (HMW-GS) and low-molecular-weight glutenin subunits (LMW-GS). For common wheat and durum wheat, the fast, easy and cheap Bradford assay was previously adapted for the quantitation of GLIA and GLUT fractions [13,14]. It has been shown that photometric analyses of protein fractions may lead to overestimated protein contents due to interfering substances [15]. Furthermore, the dye used in the Bradford assay binds to basic amino acid residues and thus, the absorbance is not only proportional to the protein content, but also depends on the amino acid sequence. Standard proteins (e.g., bovine serum albumin) were used to quantitate GLIA and GLUT fractions in durum wheat and this might lead to inaccurate results due to the low contents of basic amino acids in gluten proteins [14]. In contrast, isolated GLIA and GLUT proteins were used as reference materials for common wheat [13]. Even if the production of these reference proteins is labor-intensive, it compensates for the variation in amino acid sequences and the interferences of different buffers. The correlation of GLIA and GLUT contents in common wheat flours determined with RP-HPLC and Bradford assay revealed strong correlations (GLIA r = 0.81; GLUT r = 0.80), and thus, both methods yielded comparable results [13].

The most important advantage of the Bradford assay is that 96 samples can be measured at the same time in about one minute using a microwell plate. In comparison, one RP-HPLC run takes 30 min, and this makes this technique inconvenient for high-throughput analyses of large sample sets.

Therefore, the first aim of this study was to establish the Bradford assay for the analysis of GLIA and GLUT contents of spelt, durum wheat, emmer and einkorn. Then, GLIA and GLUT were quantitated by the Bradford assay in a sample set comprising 15 cultivars each of common wheat, spelt, durum wheat, emmer and einkorn grown at four different locations in Germany (n = 300). Finally, the relevance of genetic and environmental factors on GLIA, GLUT and gluten contents was studied, and cultivars with high GLUT content, low GLIA/GLUT ratios and presumptive good baking quality independent of environmental effects were identified. 

## 2. Materials and Methods 

### 2.1. Chemicals

All chemicals were of analytical or higher grade and purchased from VWR Merck (Darmstadt, Germany), Serva (Heidelberg, Germany), LECO (Kirchheim, Germany) or Sigma-Aldrich (Steinheim, Germany). Water was deionized by a water purification system Arium 611VF (Sartorius, Goettingen, Germany).

### 2.2. Wheat Samples

Fifteen cultivars of each wheat species common wheat, spelt, durum wheat, emmer and einkorn were cultivated by the State Plant Breeding Institute, University of Hohenheim (Germany) at four locations in Germany (Seligenstadt (SEL), Oberer Lindenhof (OLI), Hohenheim (HOH) and Eckartsweiher (EKW)). Detailed information on the growth and climatic conditions and on the parameters of the field trials have been reported by Longin et al. (2015) [1]. All cultivars and their abbreviations are listed in Table S1. Grains were dehulled in case of spelt, emmer and einkorn and milled into wholemeal flours using a cross-beater mill (Perten Instruments, Hamburg, Germany). The wholemeal flours were stored in closed bottles at room temperature in the dark for at least two weeks before analysis. 

### 2.3. Standard Determinations 

The nitrogen content of wholemeal flours was determined in triplicate by the Dumas combustion method (ICC standard 167) using a TruSpec Nitrogen Analyzer (Leco, Kirchheim, Germany). Calibration was performed with ethylenediaminetetraacetic acid and a factor of 5.7 was used to calculate the crude protein content. 

### 2.4. Quantitation of Protein Fractions

#### 2.4.1. Extraction of Gliadin and Glutenin Fractions

Fractions of albumins/globulins (ALGL), GLIA and GLUT were extracted stepwise from 100 mg of wholemeal flour according to Thanhaeuser et al. (2015) [13]. In brief, flour was extracted two times with phosphate-buffered saline (0.4 mol/L NaCl, 0.067 mol/L Na_2_HPO_4_/KH_2_PO_4_, pH 7.6) for 10 min at 22 °C (ALGL), three times with 60% (*v*/*v*) ethanol for 10 min at 22 °C (GLIA) and two times with GLUT extraction solution (50% (*v*/*v*) 1-propanol, 2 mol/L urea, 0.05 mol/L TRIS-HCl, pH 7.5, 1% (*w*/*v*) dithiothreitol (DTT)) for 30 min at 60 °C under nitrogen. The suspensions were centrifuged and the corresponding supernatants (GLIA, GLUT, respectively) were combined, diluted to 2.0 mL with the respective solvent, and filtered through a 0.45 µm membrane. Three separate extraction experiments were carried out for each flour sample. The ALGL fractions were discarded and the GLIA and GLUT fractions were analyzed by the Bradford assay. GLIA and GLUT fractions of selected samples (n = 40) were divided into two parts for both RP-HPLC analysis and Bradford assay.

#### 2.4.2. Quantitation of GLIA and GLUT Fractions by RP-HPLC

Fractions of GLIA and GLUT were analyzed by RP-HPLC according to Geisslitz et al. (2018) [11]. Injection volumes were adapted for each species and protein fraction to fit the calibration curve: GLIA of common wheat, 10 µL; GLIA of the other wheat species, 5 µL; GLUT, 20 µL.

#### 2.4.3. Bradford Assay

GLIA and GLUT fractions were diluted 1 + 4 (100 µL + 400 µL) with 60% ethanol (GLIA) and GLUT extraction solution, respectively. For calibration, isolated GLIA and GLUT of a mix of common wheat cultivars were dissolved (4 mg/mL) in the respective solution and diluted in eight steps between 0.1 mg/mL and 4.0 mg/mL as described earlier [13]. The diluted protein and the calibration solutions (20 µL) were pipetted into 96 microwell plates (transparent, F-bottom, Brand, Wertheim, Germany). Bradford reagent (200 µL, 0.01% Coomassie Brilliant Blue G250 (*w*/*v*), 4.7% ethanol (*w*/*v*), 8.5% phosphoric acid) was added. After 5 min the absorbance was measured at 595 nm in a microplate reader (Tecan Infinite 200, Maennerdorf, Switzerland). Triplicate determinations were performed for all samples. 

### 2.5. Statistical Analysis

Statistical evaluation using Pearson correlations, one-way and two-way analysis of variance (ANOVA) with Tukey’s post hoc test at a level of significance of *p* < 0.05, and principal component analysis (PCA) was performed with OriginPro 2018b (OriginLab, Northampton, MA, USA). Heritability was calculated as $h^{2}=1-\frac{\vartheta }{\sigma_{G}^{2}}$, where ϑ is the mean variance of a difference of two best linear unbiased predictors (BLUP) and $\sigma_{G}^{2}$ the genetic variance [16,17].

## 3. Results and Discussion

### 3.1. Establishment of the Bradford Assay for Spelt, Durum Wheat, Emmer and Einkorn

**Figure 1:**
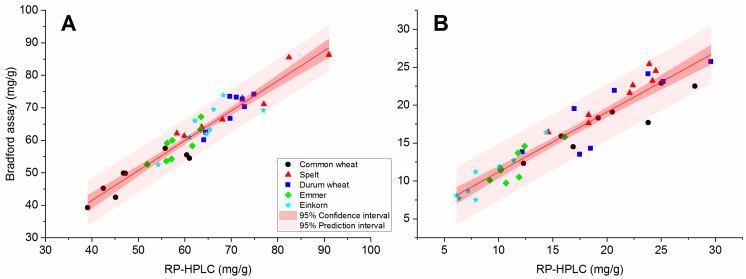
Correlation between RP-HPLC and Bradford assay for the quantitation of gliadins (**A**) and glutenins (**B**) in eight cultivars each of common wheat, durum wheat, spelt, emmer and einkorn.

GLIA and GLUT fractions isolated from a mixture of common wheat cultivars were shown to be suitable as calibrants to quantitate the respective fractions of common wheat using the Bradford assay [13]. It was unknown if the GLIA and GLUT fractions from common wheat were also suitable as calibrants for the other wheat species, because the composition of gluten protein types varies considerably [11,18]. To check this, contents of GLIA and GLUT fractions in eight cultivars of each wheat species, which were already characterized in detail for gluten protein composition and for baking quality [11], were quantitated both with the photometric Bradford assay and by RP-HPLC (detailed results in Table S2). The correlation coefficients (r) between the contents determined by RP-HPLC and by Bradford assay for individual species were r = 0.779 and higher showing a strong correlation between both methods for each wheat species (Table 1). The levels of significance were at least significant (*p* ≤ 0.05), but in most cases at least highly significant (*p* ≤ 0.01). The overall correlation coefficient for all five wheat species was very high (GLIA: r = 0.951; GLUT: r = 0.954, n = 40), with very high significance levels (*p* < 0.001) (data not shown). For the GLIA content, all data points were located within the prediction interval (Figure 1) and for the GLUT content, only one data point was outside the interval. The data points were distributed unevenly around the regression line and no trend (consistently too low or too high values for one method) was observed for each wheat species. 

The average coefficients of variation (CV) within the wheat species were higher for the Bradford assay (GLIA: 3.0–4.9%; GLUT: 3.0–8.0%) than those of the RP-HPLC analysis (GLIA: 1.0–2.1%; GLUT: 1.6–4.5%), with one exception, but in a very good acceptable range with only six CV above 8% (maximum: 13.7%). This confirmed the good reproducibility and precision of the assay as already reported for common wheat [13].

The high correlation between both methods and the low CV of the Bradford assay confirmed that the GLIA and GLUT fractions prepared from common wheat were suitable as reference materials for spelt, durum wheat, emmer and einkorn. The GLIA, GLUT and gluten (sum of GLIA and GLUT) contents were then analyzed for all samples.

### 3.2. Quantitation of GLIA, GLUT, Gluten and Total Protein Contents

**Figure 2:**
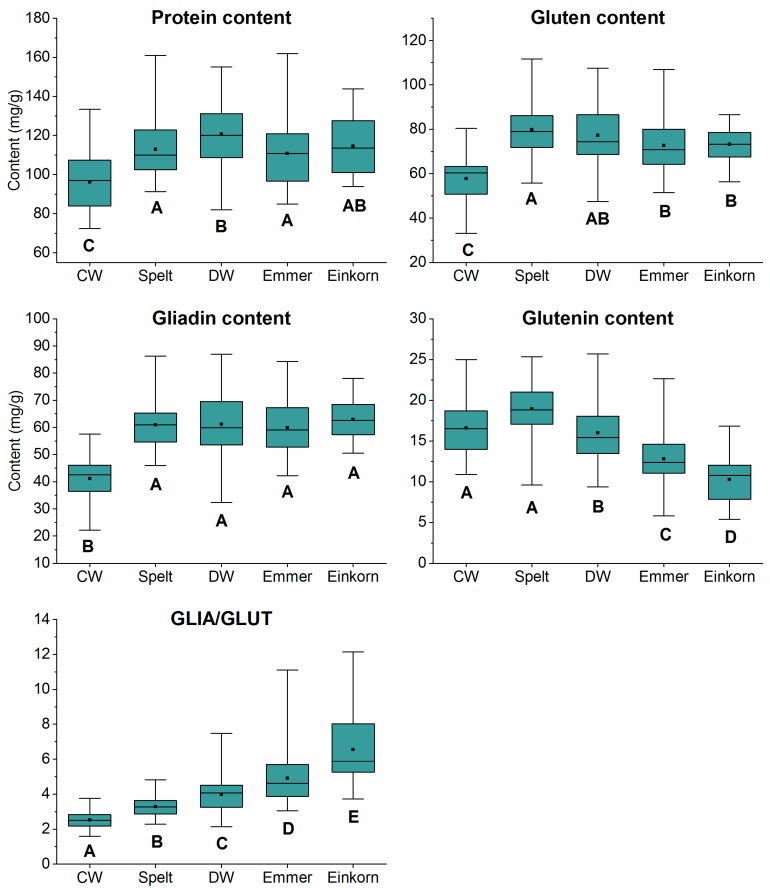
Contents of protein, gluten, gliadins, glutenins and the ratio between gliadins and glutenins (GLIA/GLUT) in common wheat (CW), spelt, durum wheat (DW), emmer and einkorn. Data are presented as median (line in the box) of 15 cultivars per wheat species cultivated at four locations (n = 60), boxes represent the interquartile range, the rectangle in the box represents the mean value, whiskers designate minima and maxima and different capital letters indicate significant differences between the wheat species (one-way analysis of variance (ANOVA), Tukey’s test at *p* < 0.05).

The determination of GLIA and GLUT contents by the Bradford assay and the total protein content by the Dumas method were performed in triplicate per sample (15 cultivars each of five wheat species grown at four different locations, n = 297). Three einkorn cultivars grown at OLI were not available because of too small yields. The mean values of triplicate determinations for GLIA, GLUT, gluten and total protein contents, and the GLIA/GLUT ratio are displayed in Table S3 for each cultivar. Figure 2 shows the distribution of the values for each wheat species (n = 60 for each box plot, i.e., 15 cultivars at four locations each). 

As expected, common wheat had the lowest protein content (mean 96.1 mg/g) compared to the other four wheat species and durum wheat the highest one (mean 120.7 mg/g). Overall, the common wheat cultivar Lear (LEA) had the lowest protein content at OLI (72.4 mg/g), whereas the emmer cultivar CC1E-04059/04 (EM8) had the highest one at OLI (161.9 mg/g). In accordance with the protein content, common wheat had the lowest GLIA (mean 41.2 mg/g) and gluten contents (mean 80.4 mg/g) compared to the other four wheats. The common wheat cultivar LEA had the lowest gluten content at OLI (33.2 mg/g), whereas the spelt cultivar Oberkulmer Rotkorn (OBR) had the highest one at SEL (111.6 mg/g). The common wheat cultivar LEA also had the lowest GLIA content at OLI (22.2 mg/g), whereas the durum wheat cultivar W-05020/01 (DU4) had the highest one at SEL (87.0 mg/g). The GLUT content decreased significantly from common wheat (mean 16.6 mg/g) and spelt (mean 19.0 mg/g) to einkorn (mean 10.3 mg/g), with durum wheat (mean 16.0 mg/g) and emmer (mean 12.8 mg/g) in between. The einkorn cultivar M-04018/03 (K08) had the lowest GLUT content at HOH (5.4 mg/g), whereas the spelt cultivar OBR had the highest one at SEL (25.4 mg/g). The differences in GLIA and GLUT contents consequently led to significant differences in the GLIA/GLUT ratios. A significant increasing trend was seen for GLIA/GLUT of the five wheat species. Common wheat had the lowest GLIA/GLUT (mean 2.5) and einkorn the highest (mean 6.5) with spelt (mean 3.3), durum wheat (mean 4.0) and emmer (mean 4.9) in between. The common wheat cultivar Colonia (COL) had the lowest GLIA/GLUT at EKW (1.6) and the einkorn cultivar M-04018/03 (K08) had the highest one at OLI (12.1). 

Usually, the gluten content corresponds to 80–90% of the protein content, but here it was between 60–80%. One reason for the lower proportion is that wholemeal flours were analyzed, and thus, more proteins of the ALGL fraction were present than in white flour. This confirmed our previous study, in which ALGL corresponded to 17–32% of the total protein content and gluten to 68–83% [11]. The variations in protein, gluten, GLIA and GLUT contents observed in the sample set are according to expectations, because the contents are known to be influenced by species and cultivars as well as growing conditions [18,19,20,21]. 

### 3.3. Nitrogen Partial Factor Productivity 

We calculated the nitrogen partial factor productivity (PFP) using the ratio of kernel yield and quantity of nitrogen fertilizer. Comparing the overall total average for each wheat species of all four locations, emmer had the highest PFP and durum wheat the lowest (Table 2). 

Due to the fact that not only high kernel yields, but also high protein contents are important in wheat, the protein yield efficiency was calculated as a ratio of protein yield, which is the product of protein content and grain yield and quantity of fertilizer. It was remarkable that the ancient wheats spelt, emmer and einkorn had higher protein yield efficiency than the modern wheats common wheat and durum wheat. Thus, regarding either only grain yield or protein yield, it appears that ancient wheats have a better potential to use nitrogen more efficiently than modern wheats, underlining their potential as alternative crops for sustainable agriculture.

Agronomic treatments including fertilization were performed according to the standard practices for the individual sites based on minimal amount of nitrogen required and local experience, as is common and has been reported earlier [19]. Thus, the fertilizer quantities were adapted to the respective needs of the wheat species and avoid lodging of spelt, emmer and einkorn (common wheat and durum wheat, 95–125 kg/ha; spelt, 60–95 kg/ha; emmer and einkorn, 0–75 kg/ha) [1], while still ensuring good comparability of the samples as has been demonstrated earlier [5,7]. 

### 3.4. Impact of Environmental Effects on GLIA, GLUT, Gluten and Total Protein Contents

**Figure 3:**
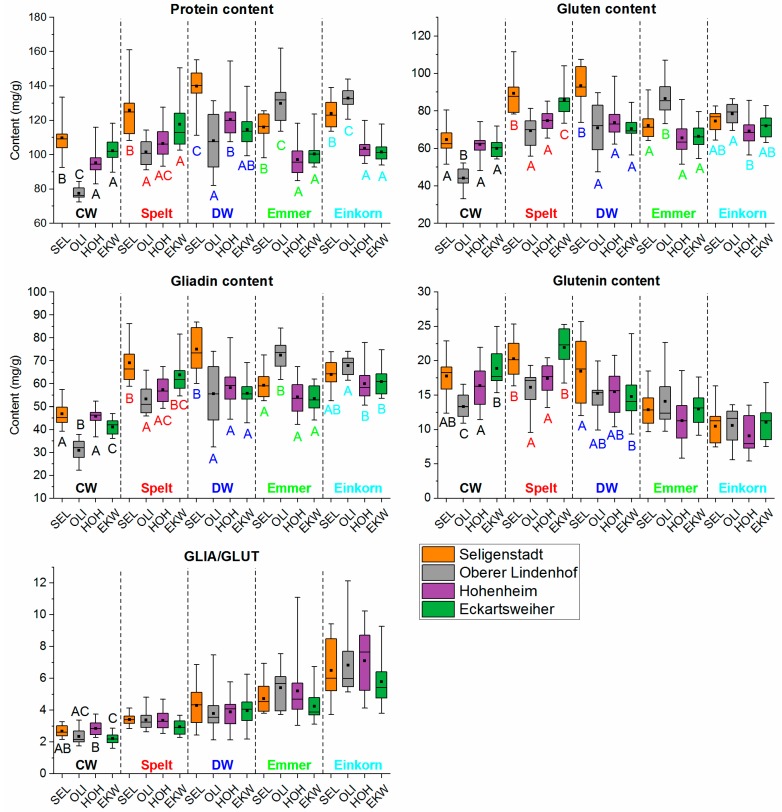
Contents of protein, gluten, gliadins, glutenins and the ratio between gliadins and glutenins (GLIA/GLUT) in common wheat (CW), spelt, durum wheat (DW), emmer and einkorn. Data are presented as median (line in the box) of 15 cultivars per wheat species and growing location (SEL, Seligenstadt; OLI, Oberer Lindenhof; HOH, Hohenheim; EKW, Eckartsweiher), boxes represent the interquartile range, the rectangle in the box represents the mean value, whiskers designate minima and maxima and different capital letters indicate significant differences between growing locations within each wheat species (one-way ANOVA, Tukey’s test at *p* < 0.05).

In contrast to Figure 2, in which all mean values of each wheat species are summarized in one box plot (n = 60 for each box plot), the mean values are divided according to the four cultivation sites (n = 15 for each box plot) in Figure 3. 

In Figure 3, the colored capital letters indicate significant differences between the four locations within the wheat species (ANOVA). To clarify further relationships between wheat species and growing location, the data were analyzed by two-way ANOVA. This provided F-values for the influence of species or location; the higher F the greater the influence. Gluten (wheat species, F = 88.8, *p* < 0.001; location, F = 20.6, *p* < 0.001; interaction, F = 15.4, *p* < 0.001), GLIA (wheat species, F = 109.4, *p* < 0.001; location, F = 20.7, *p* < 0.001; interaction F = 14.0, *p* < 0.001), GLUT (wheat species, F = 98.3, *p* < 0.001; location, F = 9.1, *p* < 0.001; interaction, F = 4.2, *p* < 0.001) and GLIA/GLUT (wheat species, F = 107.2, *p* < 0.001; location, F = 4.8, *p* = 0.003; interaction, F = 1.1, *p* = 0.393) were more influenced by the wheat species than by the growing location. In contrast, the protein content was influenced almost equally by both factors (wheat species, F = 51.6, *p* < 0.001; location, F = 51.3, *p* < 0.001; interaction, F = 23.0, *p* < 0.001). Independent of the growing location, common wheat generally had the lowest total protein, GLIA and gluten content and the lowest GLIA/GLUT compared to the other four wheat species. The protein contents of spelt, durum wheat, emmer and einkorn did not differ clearly, because the growing location had a more pronounced influence on emmer and einkorn than on common wheat, spelt and durum wheat. The mean protein contents of common wheat, spelt and durum wheat increased (OLI < HOH < EKW < SEL), but decreased for emmer and einkorn (EKW = HOH < SEL < OLI). 

In general, spelt had the highest gluten content, but no difference was observed in the gluten contents of durum wheat, emmer and einkorn. All four species had significantly higher gluten contents than common wheat. However, the gluten composition of all wheat species was significantly different. Independent of the growing location, einkorn had the lowest glutenin content, followed by emmer. These differences resulted in significant differences in GLIA/GLUT, which increased independent of the growing location from common wheat to einkorn, with spelt, durum wheat and emmer in between. The GLIA/GLUT was influenced by the growing location, as already reported due to environmental conditions (temperature) and amount and time of fertilization [20]. The location with the highest protein content (SEL) showed significantly higher amount of gliadins than EKW and, therefore, a higher GLIA/GLUT. Thus, high protein content did not consequently lead to a better ratio of GLIA/GLUT and a high amount of glutenins. These findings are in accordance with the literature [21,22,23,24].

### 3.5. Correlations between GLIA, GLUT, Gluten and Total Protein Contents and Statistics by PCA

In general, a high protein content was correlated with a high gluten (r = 0.793) and a high GLIA content (r = 0.782) considering the data from all wheat species. Higher correlations were observed within the wheat species common wheat, durum wheat and emmer (protein and gluten content: r ≥ 0.891; protein and GLIA content: r ≥ 0.821) compared to spelt and emmer (protein and gluten content: r ≥ 0.645; protein and GLIA content: r ≥ 0.641). The reasons for this difference are still unknown. Furthermore, GLIA and gluten contents showed a high correlation over all five wheat species (r = 0.946) and a high correlation of at least r ≥ 0.934 for individual wheat species. This was because GLIA accounted for 61–92% of gluten (common wheat 61–79%; spelt 70–83%; durum wheat 68–88%; emmer 75–92%; einkorn 79–92%). No correlation was observed between GLUT and protein content (r = 0.253), GLUT and GLIA content (r = 0.118) and GLUT and gluten content (r = 0.433) over all five wheat species. GLUT and gluten content were highly correlated for common wheat (r = 0.778) and weakly correlated for spelt (r = 0.662) and emmer (r = 0.596). Only for common wheat, a correlation between GLUT and protein content (r = 0.827) and GLUT and GLIA content (r = 0.578) was observed, but not for the other wheat species. This showed that the wheat species differed highly in the gluten composition and, therefore, in their GLIA/GLUT ratios. The GLIA/GLUT ratio is typically between 1.5–3.1 for common wheat and this is associated with good baking performance [12,13]. The GLIA/GLUT ratio of common wheat was confirmed in this study (1.6–3.8, mean: 2.5). Our results for eight cultivars grown at one location (spelt: 2.8–4.0; durum wheat: 2.2–5.3, emmer: 3.6–6.7, einkorn: 4.2–12.0) [11] are now expanded to four locations and 15 cultivars (spelt: 2.3–4.8, durum wheat: 2.1–7.5, emmer: 3.0–11.1, einkorn: 3.7–12.1). The high GLIA/GLUT ratios of spelt, emmer and einkorn led to poor baking performance and soft dough due to excess GLIA [1,9,10,11].

**Figure 4:**
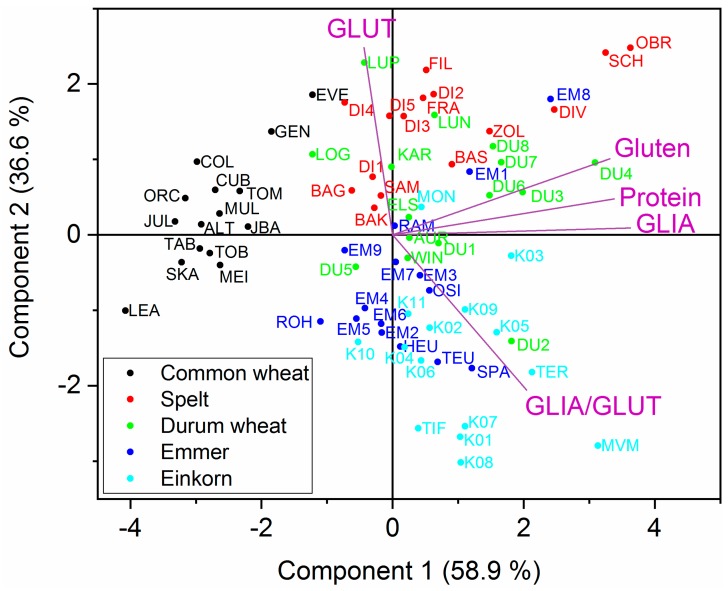
Principal component analysis biplot based on contents of gliadins (GLIA), glutenins (GLUT), gluten and protein, and ratio between gliadins and glutenins (GLIA/GLUT) using the mean value of the four locations. Abbreviations of cultivars can be found in Table S1.

Figure 4 shows the biplot of the component score and variable loadings of the PCA, which was performed with the mean values of GLIA, GLUT, gluten and protein contents and GLIA/GLUT. In Figure S1, the biplots of each individual growing location are displayed. All emmer and einkorn cultivars were located in the area of the loading of GLIA/GLUT, due to their high GLIA/GLUT ratios compared to common wheat, spelt and durum wheat. A tight clustering of common wheat cultivars in the opposite direction of GLIA, gluten and protein was observed in all biplots, because they had the lowest contents of GLIA, gluten and protein. The data points of spelt and durum wheat were positioned between the GLUT loading and the GLIA, gluten and protein loadings. This showed that spelt and durum wheat in general had the highest protein and gluten contents. With the exception of common wheat, no clear distinction of the different wheat species was possible due to partially overlapping positions in the PCA plot (Figure 4). 

### 3.6. Breeding for Optimal GLIA, GLUT, Gluten and Protein Contents

We identified a significant genetic variance for almost all wheat species regarding protein, gluten, GLUT and GLIA contents as well as the GLIA/GLUT ratio indicating that cultivars exist within each wheat species with different expressions of the respective traits. This is a prerequisite for breeding, which was fulfilled here. Furthermore, we identified a high heritability for almost all traits in all wheat species and with similar magnitude (Table 3). The heritability (h^2^) describes the amount of genetic variance relative to the total variance observed in the field, which is the sum of genetic and environmental variance for the measured traits (here: contents of protein, gluten, GLIA and GLUT and GLIA/GLUT). In general, the heritability is between 0–1 and the higher the heritability, the higher the genetic effect. With a high heritability, the choice of a specific cultivar warrants a good trait value along the production chain. As already shown for the agronomic performance [1], the heritability was higher than reported in other studies and confirmed the high quality of the field trials. Thus, our data present a robust estimation of the properties of the investigated cultivars, even though the sample set was cultivated in one year. Finally, a low GLIA/GLUT and a high GLUT content are important for good baking quality. It seems to be most promising to select for reduced GLIA/GLUT and high GLUT contents in further breeding of spelt, emmer and einkorn. 

### 3.7. Identification of Cultivars with Good Baking Performance

**Figure 5:**
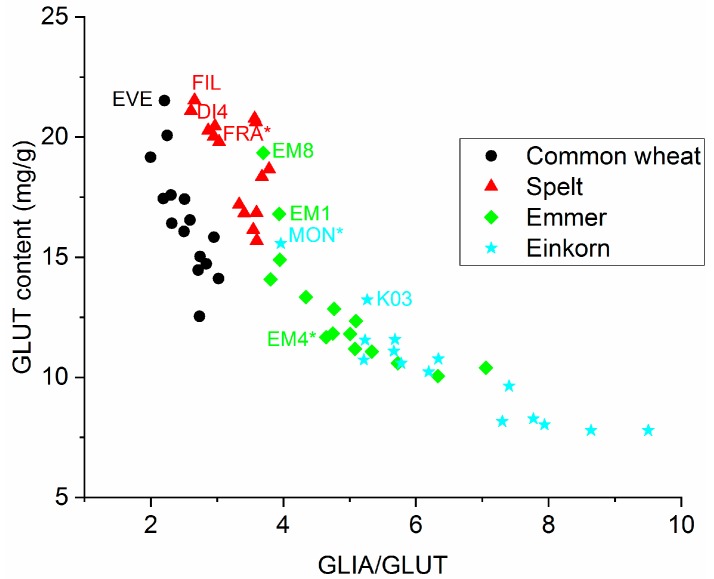
Scatter plot of glutenin (GLUT) content versus ratio between gliadins and glutenins (GLIA/GLUT) to identify cultivars with good predicted baking performance. The cultivars marked with an asterisk had already been identified as cultivars with good baking performance in our previous study [11]. Only samples with good predicted baking performance are labeled with abbreviations for better visibility. Abbreviations of cultivars are given in Table S1.

In our previous study [11], we identified one cultivar each of spelt (Franckenkorn, FRA), emmer (CC1E-04058/01, M04) and einkorn (Monlis, MON) with good baking performance and high bread volumes. GLUT content (r = 0.804) and GLIA/GLUT (r = −0.829) were defined as two important prediction parameters for bread volume. To identify cultivars with good baking performance independent of the growing location, the GLUT content was plotted versus GLIA/GLUT (mean over four locations in Figure 5 and each location in Figure S2). Durum wheat was not included, because it is mostly used for pasta making and not for bread.

The spelt cultivars Filderstolz (FIL) and D-04004/09 (DI4) had, on average, the highest GLUT and lowest GLIA/GLUT compared to the other spelt cultivars over all four locations. FIL had already been included in our previous study, but not DI4. DI4 had one of highest GLUT and one of the lowest GLIA/GLUT for three of four locations and FIL for two of four locations. The previously identified cultivar FRA was located close to FIL and DI4 and had high GLUT and low GLIA/GLUT for three of four locations. 

The emmer cultivars 9.105/06/01 (EM1) and CC1E-04059/04 (EM8) had, on average, the highest GLUT content and the lowest GLIA/GLUT over all four locations compared to the other emmer cultivars. In our previous study, we used PCA with a variety of parameters to identify cultivars with good baking performance and found only small differences within the emmer cultivars as evidenced by a tight cluster [11]. Here, the variations in GLUT and GLIA/GLUT were higher compared to our previous study, possibly due to the larger sample set with more overall variation. 

On average, the einkorn cultivars Monlis (MON) and 8.116/04 (K03) had the highest GLUT content and the lowest GLIA/GLUT over all four locations compared to the other einkorn cultivars. While K03 had not been selected previously, MON had already been identified as the einkorn cultivar giving the highest bread volume. MON had the highest GLUT content and the lowest GLIA/GLUT at three of four locations and is, therefore, one of the einkorn cultivars with the best predicted baking performance. However, the yield was very low at OLI leaving no material available for analysis. For a successful cultivar, both high yield and good baking quality are required, so that MON appears to be a suitable candidate for further improvement through breeding.

## 4. Conclusions

The Bradford assay adapted for wheat proteins is a suitable tool to quantitate quality-related proteins and protein fractions of common wheat, spelt, durum wheat, emmer and einkorn. The method is easy to use and allows routine analysis of a high number of samples in a short time. Furthermore, we used isolated GLIA and GLUT fractions from common wheat as reference material for calibration, which were suitable for all five wheat species. This compensated one big disadvantage of the Bradford assay, because the absorbance and the dye binding depends on the amino acid sequence of the analyzed proteins. 

For the first time, the protein and gluten contents and the gluten protein composition (GLIA and GLUT) of 15 cultivars each of common wheat, spelt, durum wheat, emmer and einkorn, which were grown at four different locations in Germany, were analyzed. To our knowledge, this is the first study comparing the five wheat species cultivated at four locations. Thus, this study represents a robust dataset for all five wheat species that shows the effect of the wheat species, but also the effects of different locations on protein and gluten contents and gluten protein composition. 

The key protein parameters related with baking quality are high GLUT content and a relatively low GLIA/GLUT ratio. This enables the identification of cultivars within wheat species with predicted good baking performance independent of the growing location. To improve dough and baking properties of einkorn and emmer, breeding programs should select for low GLIA/GLUT and high GLUT content. Furthermore, common wheat had the lowest protein, gluten and GLIA contents, which is actually contrary to consumer expectations who typically think that ancient wheats have lower gluten contents. A side effect of increasing the cultivation of spelt, emmer and einkorn is their higher nitrogen PFP compared to common wheat. This makes these ancient wheats interesting crops for a more sustainable agriculture. 

## Figures and Tables

**Table 1:** Correlation coefficients (r) and levels of significance (*p*) between reversed-phase high-performance liquid chromatography (RP-HPLC) and Bradford assay for the quantitation of gliadins (GLIA) and glutenins (GLUT) in common wheat, spelt, durum wheat, emmer and einkorn.

Wheat Species	Number of Samples	GLIA		GLUT	
		r ^1^	*p* ^2^	r ^1^	*p* ^2^
Common wheat	8	0.898	0.002 **	0.918	0.001 ***
Spelt	8	0.956	<0.001 ***	0.960	<0.001 ***
Durum wheat	8	0.885	0.003 **	0.862	0.006 **
Emmer	8	0.836	0.010 **	0.812	0.014
Einkorn	8	0.779	0.023 *	0.944	<0.001 ***

^1^ r < 0.54 = no correlation; 0.54–0.66 = weak correlation; 0.67–0.78 = medium correlation; >0.78 = strong correlation according to Thanhaeuser et al. (2015) [13]. ^2^ * significant (*p* ≤ 0.05); ** highly significant (*p* ≤ 0.01); *** very highly significant (*p* ≤ 0.001).

**Table 2:** Nitrogen partial factor productivity (PFP) and protein yield efficiency (PYE) of common wheat, spelt, durum wheat, emmer and einkorn. PFP is calculated as ratio of kernel yield (in decitonnes (dt) per hectare (ha), i.e., 10,000 m^2^) and quantity of nitrogen fertilizer (in kg of nitrogen (N)). PYE is calculated as ratio of protein yield, which is the product of protein content (in %) and kernel yield, and quantity of fertilizer. Kernel yield, quantity of nitrogen fertilizer and protein content are means over all cultivars at all four locations within the wheat species.

Wheat Species	PFP	Kernel Yield (dt/ha) ^1^	Nitrogen Fertilizer	Protein Content	PYE
Unit	(dt/kg × N)	(dt/ha) ^1^	(kg × N/ha) ^1^	(%)	(t/kg × N)
Common wheat	0.7	79.8	115	9.6	0.67
Spelt	0.7	50.4	75	11.3	0.76
Durum wheat	0.5	60.9	115	12.5	0.66
Emmer	1.2	36.2	30	11.1	1.34
Einkorn	0.9	26.9	30	11.6	1.04

^1^ As reported by Longin et al. [1].

**Table 3:** Heritability (h^2^) of the contents of protein, gluten, gliadins (GLIA), and glutenins (GLUT), and the ratio between gliadins and glutenins (GLIA/GLUT), based on 15 cultivars of each wheat species grown at four locations.

Wheat Species	Protein	Gluten	GLIA	GLUT	GLIA/GLUT
Common wheat	0.88	0.90	0.83	0.93	0.82
Spelt	0.94	0.91	0.91	0.78	0.68
Durum wheat	0.77	0.82	0.80	0.88	0.94
Emmer	0.85	0.88	0.82	0.89	0.71
Einkorn	0.92	0.78	0.78	0.83	0.78

## Supplementary Materials

The following are available online at https://www.mdpi.com/2304-8158/8/9/409/s1, Table S1: Overview of all 75 samples of common wheat, spelt, durum wheat, emmer and einkorn and their abbreviations, Table S2: Gliadin and glutenin fractions extracted by modified Osborne fractionation and quantitated both by reversed phase high-performance liquid chromatography (RP-HPLC) and by photometric Bradford assay. CV, coefficient of variation, Table S3: Contents of gliadins, glutenins, gluten and protein, and ratio between gliadins and glutenins (GLIA/GLUT) determined by photometric Bradford assay of 15 cultivars per wheat species (common wheat, spelt, durum wheat, emmer and einkorn) cultivated at four different locations, Figure S1: Biplot of principle component analysis (PCA) of gliadins (GLIA), glutenins (GLUT), gluten, protein content and ratio between GLIA and GLUT (GLIA/GLUT) of the five wheat species common wheat, spelt, durum wheat, emmer and einkorn for the four locations Seligenstadt, Oberer Lindenhof, Hohenheim and Eckartsweiher, Figure S2: Scatterplot of glutenin content (GLUT) versus ratio of gliadins and glutenins (GLIA/GLUT) of common wheat, spelt, emmer and einkorn for the four locations Seligenstadt, Oberer Lindenhof, Hohenheim and Eckartsweiher for the identification of cultivars with predicted good baking performance.
