# Supplementary material for: Comparative Study on Gluten Protein Composition of Ancient (Einkorn, Emmer and Spelt) and Modern Wheat Species (Durum and Common Wheat)
Source: Foods. 2019 Sep 12;8(9):409. doi: 10.3390/foods8090409 (PMC6769531; doi:10.3390/foods8090409)
Supplement: Supplementary file 1 [file foods-08-00409-s001.pdf]

Supplementary Materials

Comparative Study on Gluten Protein Composition of Ancient (Einkorn, Emmer and Spelt) and Modern Wheat Species (Durum and Common Wheat)

Sabrina Geisslitz [1], C. Friedrich H. Longin [2], Katharina A. Scherf [1,3]*, Peter Koehler [4]

[1] Leibniz-Institute for Food Systems Biology at the Technical University of Munich, Lise-Meitner-Strasse 34, 85354 Freising, Germany

[2] State Plant Breeding Institute, University of Hohenheim, 70599 Stuttgart, Germany

[3] Department of Bioactive and Functional Food Chemistry, Institute of Applied Biosciences, Karlsruhe Institute of Technology (KIT), Adenauerring 20 a, 76131 Karlsruhe, Germany

[4] biotask AG, Schelztorstrasse 54-56, 73728 Esslingen am Neckar, Germany

Table S1

Overview of all 75 samples of common wheat, spelt, durum wheat, emmer and einkorn and their abbreviations

**Common wheat**		**Spelt**		**Durum wheat**		**Emmer**		**Einkorn**	
Altigo	ALT	Badengold	BAG	Auradur	AUR	9.105/06/01*	EM1	8.103/04*	K01
Colonia	COL	Badenkrone	BAK	Elsadur	ELS	9.118/05/01*	EM2	8.108/04**	K02
Cubus	CUB	Badenstern	BAS	Karur	KAR	9.199/06/01*	EM3	8.116/04*	K03
Event	EVE	D-04004/03*	DI1	Logidur	LOG	CC1E-04058/01*	EM4	M-03021/03*	K04
Genius	GEN	D-04004/04*	DI2	Lunadur	LUN	CC1E-04059/01*	EM5	M-04003/01*	K05
JB Asano	JBA	D-04004/08*	DI3	Lupidur *	LUP	CC1E04059/02*	EM6	M-04018/01*	K06
Julius	JUL	D-04004/09*	DI4	W-05005/02*	DU1	CC1E04059/03*	EM7	M-04018/02*	K07
Lear	LEA	D-05015/03*	DI5	W-05006/01*	DU2	CC1E-04059/04*	EM8	M-04018/03*	K08
Meister	MEI	Divimar	DIV	W-05018/04*	DU3	E-07086/01*	EM9	M-04033/01*	K09
Mulan	MUL	Filderstolz	FIL	W-05020/01*	DU4	Heuholzer Kolben	HEU	M-04033/03*	K10
Orcas	ORC	Franckenkorn	FRA	W-05024/01*	DU5	Osiris	OSI	M-07006/01*	K11
Skalmeje	SKA	Oberkulmer Rotkorn	OBR	W-05025/01*	DU6	Ramses	RAM	Monlis	MON
Tabasco	TAB	Samir	SAM	W-05026/03*	DU7	Roter Heidfelder	ROH	MV Menket	MVM
Tobak	TOB	Schwabenkorn	SCH	W-05029/02*	DU8	Späth’s Albjuwel	SPA	Terzino	TER
Tommi	TOM	Zollernspelz	ZOL	Wintergold	WIN	Teutonia	TEU	Tifi	TIF

* = cultivar from the State Plant Breeding Institute (University of Hohenheim)

Table S2

Gliadin and glutenin fractions extracted by modified Osborne fractionation and quantitated both by reversed phase high-performance liquid chromatography (RP-HPLC) and by photometric Bradford assay. CV, coefficient of variation

	**Gliadin content (mg/g)[a]**		**Glutenin content (mg/g)[a]**	
	**RP-HPLC**	**Bradford**	**RP-HPLC**	**Bradford**
**Common wheat**				
Mulan	46.8 ± 1.4	49.9 ± 1.5	20.5 ± 0.6	19.1 ± 1.3
Tommi	47.2 ± 0.3	49.8 ± 1.6	23.8 ± 0.8	17.7 ± 0.8
Tabasco	42.5 ± 0.2	45.2 ± 1.4	16.9 ± 0.0	14.5 ± 0.6
Event	61.0 ± 2.3	54.5 ± 2.3	28.1 ± 0.4	22.5 ± 1.8
JB Asano	60.4 ± 0.1	55.5 ± 0.6	19.2 ± 0.6	18.3 ± 0.9
Lear	39.1 ± 0.7	39.3 ± 1.4	12.3 ± 0.3	12.3 ± 0.8
Genius	55.8 ± 0.8	57.5 ± 1.4	25.0 ± 0.8	22.9 ± 1.2
Tobak	45.1 ± 0.5	42.4 ± 1.5	15.8 ± 0.9	15.9 ± 0.3
CV (%)	1.5	3.0	2.8	5.2
**Spelt**				
Badenkrone	58.3 ± 0.3	62.1 ± 1.6	14.6 ± 0.4	16.4 ± 0.9
Franckenkorn	63.6 ± 0.3	64.1 ± 1.5	22.4 ± 0.3	22.6 ± 1.1
Zollernspelz	77.0 ± 0.8	71.1 ± 3.4	22.1 ± 0.6	21.6 ± 1.9
Oberkulmer	91.0 ± 0.7	86.3 ± 3.2	23.9 ± 2.2	25.4 ± 0.8
Badenstern	72.4 ± 0.9	72.9 ± 1.1	18.3 ± 0.2	17.6 ± 1.2
Badengold	59.9 ± 0.9	61.3 ± 2.4	18.3 ± 0.4	18.7 ± 0.9
Schwabenkorn	82.4 ± 1.9	85.5 ± 2.8	24.5 ± 1.2	24.5 ± 0.6
Filderstolz	68.1 ± 0.3	66.4 ± 3.2	24.2 ± 0.4	23.2 ± 1.1
CV (%)	1.6	3.4	1.6	5.2
**Durum wheat**				
Auradur	72.4 ± 0.8	72.6 ± 0.4	17.5 ± 0.5	13.5 ± 0.1
Logidur	69.8 ± 1.4	66.7 ± 2.2	23.8 ± 0.5	24.1 ± 0.7
Wintergold	69.7 ± 4.4	73.5 ± 4.5	18.5 ± 0.3	14.3 ± 0.5
Lunadur	74.9 ± 1.1	74.1 ± 1.8	25.2 ± 0.4	23.1 ± 0.2
Elsadur	71.1 ± 0.6	73.2 ± 1.3	17.0 ± 0.7	19.5 ± 0.4
Karur	72.9 ± 0.2	70.3 ± 3.6	20.7 ± 0.6	21.9 ± 0.4
Lupidur	64.5 ± 2.4	62.4 ± 4.0	29.6 ± 0.0	25.7 ± 2.0
W-05005/02	64.1 ± 0.7	60.1 ± 2.6	12.2 ± 0.6	13.8 ± 0.7
CV (%)	2.1	3.7	2.5	3.0
**Emmer**				
Osiris	63.4 ± 1.3	63.4 ± 3.8	11.9 ± 0.3	10.5 ± 0.8
Ramses	56.2 ± 0.2	59.1 ± 3.0	12.4 ± 0.2	14.6 ± 1.7
Heuholzer Kolben	63.5 ± 0.8	67.2 ± 2.0	10.7 ± 0.2	9.7 ± 0.4
Teutonia	61.6 ± 0.4	58.3 ± 2.1	9.2 ± 0.7	10.1 ± 0.9
CC1E-04058/01	57.3 ± 0.7	60.0 ± 2.8	16.1 ± 0.2	15.8 ± 0.6
CC1E-04059/01	56.0 ± 0.1	53.6 ± 1.6	10.2 ± 0.4	11.4 ± 0.7
Späths Albjuwel	57.1 ± 0.5	54.3 ±3.9	10.2 ± 0.3	11.6 ± 1.6
9.105/06/01	51.9 ± 0.8	52.6 ± 3.5	11.8 ± 0.4	13.7 ± 1.1
CV (%)	1.0	4.9	3.1	8.0
**Einkorn**				
Tifi	65.4 ± 1.8	63.2 ± 3.4	7.9 ± 0.2	7.5 ± 0.5
Terzino	68.3 ± 1.2	73.9 ± 2.3	7.2 ± 0.1	8.7 ± 0.9
Monlis	61.0 ± 2.0	60.9 ± 3.4	14.4 ± 0.8	16.4 ± 0.3
M-04018/01	64.7 ± 1.5	61.9 ± 2.5	6.1 ± 0.5	8.1 ± 0.3
8.108/04	62.2 ± 0.8	66.0 ± 1.0	7.9 ± 0.2	11.2 ± 0.1
M04033/03	66.2 ± 0.4	69.5 ± 2.9	11.4 ± 0.6	12.7 ± 0.4
Mv Menket	76.9 ± 0.7	69.2 ± 2.4	6.4 ± 0.3	7.7 ± 0.4
M07006/01	54.3 ± 0.6	52.5 ± 2.4	10.1 ± 0.4	11.9 ± 0.4
CV (%)	1.8	4.0	4.5	4.3

Table S3

Contents of gliadins, glutenins, gluten and protein, and ratio between gliadins and glutenins (GLIA/GLUT) determined by photometric Bradford assay of 15 cultivars per wheat species (common wheat, spelt, durum wheat, emmer and einkorn) cultivated at four different locations[a]

Name[b]	Gliadins (mg/g)				Glutenins (mg/g)				Gluten (mg/g)				Protein (%)				GLIA/GLUT[c]			
	SEL	OLI	HOH	EKW	SEL	OLI	HOH	EKW	SEL	OLI	HOH	EKW	SEL	OLI	HOH	EKW	SEL	OLI	HOH	EKW
Common wheat																				
MUL	49.1	32.1	46.2	42.0	19.1	11.9	15.7	19.5	68.2	44.0	61.9	61.5	10.9	7.7	9.6	10.0	2.6	2.7	2.9	2.2
TOM	49.8	34.9	44.9	44.9	17.7	15.0	17.0	20.0	67.4	49.9	61.9	65.0	10.9	8.1	9.8	10.7	2.8	2.3	2.6	2.2
TAB	45.2	37.2	43.3	37.6	14.5	12.8	15.7	17.1	59.6	50.0	59.0	54.7	10.0	8.4	9.2	9.5	3.1	2.9	2.8	2.2
EVE	54.5	35.7	52.4	46.9	22.5	16.6	21.9	25.0	77.1	52.2	74.3	71.9	13.3	8.4	11.6	11.8	2.4	2.2	2.4	1.9
SKA	43.6	32.3	41.5	38.3	16.6	11.9	13.1	16.3	60.2	44.3	54.6	54.5	10.4	7.7	8.7	9.8	2.6	2.7	3.2	2.4
ALT	42.3	28.1	45.4	42.8	19.5	13.4	13.6	17.7	61.9	41.4	59.1	60.5	11.2	7.6	9.4	10.2	2.2	2.1	3.3	2.4
JBA	55.5	37.8	47.4	43.8	18.3	11.2	16.4	17.3	73.8	49.0	63.8	61.2	11.5	7.5	9.2	9.9	3.0	3.4	2.9	2.5
JUL	43.1	25.8	46.2	38.0	18.4	13.4	16.3	17.5	61.5	39.2	62.5	55.6	10.9	7.5	8.8	9.3	2.3	1.9	2.8	2.2
LEA	39.2	22.2	36.7	39.1	12.3	11.0	11.4	15.4	51.6	33.2	48.1	54.4	9.3	7.2	8.3	9.0	3.2	2.0	3.2	2.5
CUB	45.8	27.8	46.4	42.0	17.3	14.3	17.3	21.4	63.1	42.1	63.7	63.4	11.0	7.6	9.5	11.3	2.6	1.9	2.7	2.0
MEI	47.9	29.2	50.6	41.6	14.7	10.9	13.5	17.4	62.5	40.1	64.1	59.0	10.7	7.6	9.6	10.3	3.3	2.7	3.8	2.4
ORC	43.8	28.7	43.9	36.1	18.3	13.3	18.5	19.7	62.0	42.1	62.4	55.8	10.9	7.5	9.4	9.9	2.4	2.2	2.4	1.8
COL	44.4	26.4	45.8	36.2	18.9	15.1	19.9	22.7	63.3	41.5	65.7	58.9	11.2	7.6	10.0	10.4	2.3	1.8	2.3	1.6
GEN	57.5	32.5	49.8	42.0	22.9	16.2	20.2	21.0	80.4	48.7	70.0	63.0	12.1	8.1	10.6	11.0	2.5	2.0	2.5	2.0
TOB	49.1	33.4	46.9	44.8	15.9	12.7	14.7	15.6	58.2	46.1	61.6	60.4	10.1	7.8	9.1	10.3	2.7	2.6	3.2	2.9
Mean[d]	46.9	30.9	45.8	41.1	17.8	13.3	16.3	18.9	64.7	44.3	62.2	60.0	11.0	7.7	9.5	10.2	2.7	2.4	2.9	2.2
CV (%)[e]	3.8	6.0	3.0	7.1	4.7	6.4	4.8	3.8	-	-	-	-	0.7	1.1	0.7	0.9	-	-	-	-
Spelt																				
BAG	61.3	57.5	49.2	54.6	18.7	12.5	17.5	18.7	80.0	70.0	66.7	73.3	11.0	9.6	9.6	10.5	3.3	4.6	2.8	2.9
BAK	62.1	47.3	55.6	61.4	16.4	14.3	15.3	16.7	78.4	61.6	70.9	78.2	10.8	9.4	10.1	10.6	3.8	3.3	3.6	3.7
BAS	72.8	47.2	58.2	64.5	17.6	14.1	16.3	19.4	90.4	61.3	74.6	83.9	12.6	9.4	10.8	11.7	4.1	3.3	3.6	3.3
DI1	61.9	45.9	52.2	64.1	17.0	15.7	13.2	22.9	78.9	61.6	65.4	87.0	11.1	9.4	9.3	11.3	3.6	2.9	4.0	2.8
DI2	71.4	50.0	59.2	60.2	20.8	17.1	19.3	24.7	92.1	67.0	78.5	84.9	12.5	10.1	10.2	11.0	3.4	2.9	3.1	2.4
DI3	66.7	57.0	57.3	57.9	21.1	17.6	18.8	21.8	87.7	74.6	76.1	79.7	11.9	10.4	10.2	10.3	3.2	3.2	3.0	2.7

Name[b]	Gliadins (mg/g)				Glutenins (mg/g)				Gluten (mg/g)				Protein (%)				GLIA/GLUT[c]			
	SEL	OLI	HOH	EKW	SEL	OLI	HOH	EKW	SEL	OLI	HOH	EKW	SEL	OLI	HOH	EKW	SEL	OLI	HOH	EKW
DI4	58.9	50.9	50.6	57.6	19.8	19.3	20.0	25.3	78.7	70.2	70.6	82.9	11.2	9.6	9.4	10.5	3.0	2.6	2.5	2.3
DI5	61.2	53.4	56.8	61.8	18.1	18.0	19.2	24.8	79.3	71.5	76.0	86.6	12.3	9.7	10.0	11.1	3.4	3.0	3.0	2.5
DIV	82.9	54.7	66.6	73.2	20.1	17.6	14.2	22.8	103.0	72.3	80.8	96.0	13.6	10.8	11.3	13.3	4.1	3.1	4.7	3.2
FIL	66.4	47.5	53.7	60.6	23.2	17.4	20.4	25.1	89.6	65.0	74.1	85.7	13.0	10.3	10.6	12.1	2.9	2.7	2.6	2.4
FRA	64.0	50.5	55.6	62.5	22.6	19.1	19.3	20.2	86.6	69.5	74.8	82.7	12.8	10.5	10.8	11.9	2.8	2.6	2.9	3.1
OBR	86.3	62.0	67.5	78.3	25.4	17.1	17.7	22.3	111.6	79.1	85.2	100.6	16.1	11.3	12.8	15.1	3.4	3.6	3.8	3.5
SAM	63.0	46.2	52.2	54.6	18.2	9.6	15.8	21.0	81.2	55.7	68.1	75.6	12.5	9.1	10.6	10.7	3.5	4.8	3.3	2.6
SCH	85.5	63.9	64.8	81.7	24.5	17.4	18.8	22.4	110.0	81.3	83.6	104.1	14.4	11.4	12.3	14.3	3.5	3.7	3.4	3.6
ZOL	71.1	65.8	61.9	65.7	21.5	15.3	15.7	20.9	92.7	81.0	77.7	86.6	13.0	11.4	11.4	12.4	3.3	4.3	3.9	3.1
Mean[d]	69.0	53.3	57.4	63.9	20.3	16.1	17.4	21.9	89.3	69.5	74.9	85.8	12.6	10.2	10.6	11.8	3.4	3.4	3.4	2.9
CV (%)[e]	3.1	4.5	4.6	3.1	4.6	6.0	4.9	4.2	-	-	-	-	0.7	1.0	0.9	0.6	-	-	-	-
Durum wheat																				
AUR	72.6	42.1	59.2	59.7	13.5	12.8	16.5	14.1	86.0	55.0	75.8	73.8	13.7	9.0	12.1	11.9	5.4	3.3	3.6	4.2
DU1	60.1	71.8	51.4	56.9	13.8	15.4	11.8	14.0	73.9	87.2	63.2	70.9	14.0	8.2	11.3	10.4	4.3	4.7	4.4	4.1
DU2	82.8	74.2	60.0	58.6	12.0	9.9	10.4	9.4	94.8	84.1	70.4	68.0	13.6	8.6	13.1	10.7	6.9	7.5	5.8	6.3
DU3	84.7	67.4	65.4	57.9	18.8	15.7	16.1	11.5	103.5	83.1	81.4	69.3	14.6	10.4	12.4	11.9	4.5	4.3	4.1	5.0
DU4	87.0	62.6	80.0	67.8	17.1	13.6	18.5	15.6	104.0	76.1	98.5	83.4	15.1	9.5	12.1	11.9	5.1	4.6	4.3	4.3
DU5	64.5	55.1	50.1	43.7	12.6	15.5	12.3	12.7	77.1	70.6	62.3	56.4	14.0	10.1	11.3	10.8	5.1	3.5	4.1	3.4
DU6	84.5	63.5	62.8	54.9	18.0	15.4	15.2	13.5	102.5	78.9	78.1	68.4	15.5	9.3	11.0	12.7	4.7	4.1	4.1	4.1
DU7	84.7	56.7	63.8	69.2	22.8	15.5	14.5	15.4	107.5	72.1	78.3	84.5	13.0	12.8	10.9	12.1	3.7	3.7	4.4	4.5
DU8	83.9	69.9	60.5	55.2	20.3	19.7	14.4	16.5	104.2	89.6	74.9	71.6	12.9	12.2	11.8	10.8	4.1	3.5	4.2	3.3
ELS	73.2	41.3	56.3	55.5	19.5	15.2	12.5	12.8	92.7	56.5	68.8	68.3	14.7	12.3	12.9	11.4	3.8	2.7	4.5	4.3
KAR	70.3	51.7	53.2	53.2	21.9	15.3	16.9	15.6	92.2	67.0	70.0	68.8	15.0	11.1	15.5	14.0	3.2	3.4	3.1	3.4
LOG	66.7	32.3	53.7	42.9	24.1	15.1	17.7	17.1	90.9	47.4	71.4	60.0	11.1	12.8	10.8	9.9	2.8	2.1	3.0	2.5
LUN	74.1	55.5	53.5	55.5	23.1	17.3	19.4	18.0	97.2	72.8	72.9	73.6	14.1	12.0	12.5	10.9	3.2	3.2	2.8	3.1
LUP	62.4	44.1	44.5	52.2	25.7	20.0	20.8	24.0	88.1	64.1	65.3	76.2	14.7	10.8	12.0	11.6	2.4	2.2	2.1	2.2
WIN	73.5	47.1	60.1	54.2	14.3	12.2	15.6	11.6	87.8	59.3	75.7	65.8	13.8	13.1	11.4	11.0	5.1	3.9	3.8	4.7
Mean[d]	75.0	55.7	58.3	55.8	18.5	15.2	15.5	14.8	93.5	70.9	73.8	70.6	14.0	10.8	12.1	11.5	4.3	3.8	3.9	4.0
CV (%)[e]	3.8	4.7	3.6	4.3	3.8	4.7	5.1	5.8	-	-	-	-	0.7	0.9	0.7	0.7	-	-	-	-

Name[b]	Gliadins (mg/g)				Glutenins (mg/g)				Gluten (mg/g)				Protein (%)				GLIA/GLUT[c]			
	SEL	OLI	HOH	EKW	SEL	OLI	HOH	EKW	SEL	OLI	HOH	EKW	SEL	OLI	HOH	EKW	SEL	OLI	HOH	EKW
Emmer																				
HEU	67.2	70.2	51.4	52.8	9.7	11.5	10.2	11.0	76.9	81.7	61.6	63.9	11.6	12.0	8.9	9.6	6.9	6.1	5.1	4.8
EM1	60.0	76.9	67.3	59.1	15.8	20.6	15.4	15.3	75.8	97.5	82.7	74.4	12.4	13.6	10.7	10.9	3.8	3.7	4.4	3.9
EM2	53.6	70.0	54.5	55.2	11.4	11.7	8.8	12.4	65.0	81.7	63.3	67.6	11.2	12.1	9.2	9.5	4.7	6.0	6.2	4.4
EM3	56.0	74.4	59.5	52.0	13.6	12.4	11.4	14.0	69.6	86.8	70.9	66.0	11.8	13.2	10.5	10.0	4.1	6.0	5.2	3.7
EM4	60.5	66.1	53.7	44.1	13.7	11.6	11.4	10.5	74.2	77.7	65.1	54.6	11.7	12.0	9.6	9.3	4.4	5.7	4.7	4.2
EM5	52.6	73.6	48.0	54.0	11.6	9.7	11.3	14.6	64.2	83.3	59.3	68.6	9.8	11.7	8.8	10.0	4.5	7.6	4.3	3.7
EM6	54.3	67.7	48.4	51.9	11.6	11.1	8.5	13.6	65.9	78.8	56.9	65.5	11.9	13.2	9.4	10.2	4.7	6.1	5.7	3.8
EM7	52.6	76.8	53.2	49.4	13.7	16.1	11.1	12.5	66.3	92.9	64.2	61.9	11.3	14.3	9.9	9.7	3.8	4.8	4.8	4.0
EM8	72.6	84.3	67.5	61.9	18.5	22.7	18.6	17.7	91.1	107.0	86.0	79.6	12.5	16.2	11.8	12.4	3.9	3.7	3.6	3.5
EM9	56.5	64.1	47.7	46.0	14.7	16.2	11.8	13.5	71.2	80.4	59.5	59.5	11.4	12.8	9.5	9.5	3.8	3.9	4.0	3.4
OSI	63.4	74.0	46.0	59.6	10.4	11.4	15.1	12.5	73.8	85.4	61.1	72.1	12.4	13.2	9.9	10.6	6.1	6.5	3.0	4.8
RAM	59.1	70.0	54.5	51.0	14.6	18.4	13.5	13.1	73.6	88.4	68.0	64.1	11.6	13.4	9.7	10.1	4.0	3.8	4.1	3.9
ROH	60.2	61.8	42.2	47.0	10.9	11.5	9.3	15.0	71.2	73.3	51.5	62.0	10.7	11.5	8.5	9.3	5.5	5.4	4.6	3.1
SPA	63.1	75.5	64.5	61.7	12.8	13.8	5.8	9.1	75.9	89.3	70.3	70.8	12.5	11.4	10.2	10.1	4.9	5.5	11.1	6.7
TEU	58.3	81.9	55.0	56.9	10.1	12.6	7.4	10.1	68.4	94.5	62.4	67.0	11.3	14.2	8.9	9.4	5.8	6.5	7.4	5.6
Mean[d]	59.3	72.5	54.2	53.5	12.9	14.1	11.3	12.5	72.2	86.6	65.5	66.5	11.6	13.0	9.7	10.0	4.7	5.4	5.2	4.2
CV (%)[e]	3.8	3.2	3.5	3.4	7.3	6.9	5.9	4.9	-	-	-	-	0.9	0.8	1.0	0.8	-	-	-	-
Einkorn																				
TIF	63.2	62.2	54.7	54.6	7.5	7.3	9.3	8.5	70.7	69.5	64.0	63.1	11.6	13.1	9.5	9.8	8.5	8.5	5.9	6.4
TER	73.8	70.4	68.5	64.3	8.7	10.1	7.8	11.9	82.5	80.5	76.3	76.3	13.1	14.4	10.9	10.5	8.5	6.9	8.8	5.4
MON	60.9	-	59.1	63.9	16.4	-	13.5	16.8	77.2	-	72.6	80.7	13.1	-	10.3	10.9	3.7	-	4.4	3.8
K01	61.9	61.6	63.6	65.9	8.1	9.1	7.3	7.6	69.9	70.7	70.9	73.5	12.6	12.1	10.0	9.7	7.7	6.7	8.7	8.6
K02	65.9	65.7	58.4	53.7	11.2	12.8	8.0	12.4	77.2	78.5	66.3	66.1	12.3	13.6	9.8	9.8	5.9	5.1	7.3	4.3
K03	69.5	72.3	69.1	67.6	12.7	13.6	13.2	13.5	82.1	85.9	82.3	81.1	13.2	14.2	10.6	10.5	5.5	5.3	5.3	5.0
MVM	69.2	-	78.0	74.8	7.7	-	7.6	8.1	76.9	-	85.7	82.9	13.9	-	12.0	11.8	9.0	-	10.2	9.3
K04	52.5	61.7	60.5	62.5	11.9	11.6	7.9	10.9	64.4	73.3	68.5	73.5	11.6	12.4	10.1	9.4	4.4	5.3	7.7	5.7
K05	69.0	74.0	62.4	61.0	11.5	12.5	7.8	11.3	80.5	86.6	70.2	72.3	12.9	13.7	11.1	10.4	6.0	5.9	8.0	5.4
K06	63.9	70.4	50.5	54.5	11.9	11.6	5.9	11.6	75.7	82.0	56.4	66.1	11.8	13.1	9.9	9.8	5.4	6.1	8.6	4.7

Name[b]	Gliadins (mg/g)				Glutenins (mg/g)				Gluten (mg/g)				Protein (%)				GLIA/GLUT[c]			
	SEL	OLI	HOH	EKW	SEL	OLI	HOH	EKW	SEL	OLI	HOH	EKW	SEL	OLI	HOH	EKW	SEL	OLI	HOH	EKW
K07	70.1	66.3	55.0	58.5	7.4	7.8	7.1	10.8	77.6	74.2	62.1	69.3	12.1	13.1	10.5	10.1	9.4	8.5	7.8	5.4
K08	65.9	68.3	51.7	57.3	12.6	5.6	5.4	7.5	78.6	73.9	57.1	64.9	12.3	12.8	10.3	10.1	5.2	12.1	9.6	7.6
K09	64.3	72.0	57.3	62.1	8.5	12.8	12.0	13.0	72.9	84.8	69.4	75.0	12.8	13.7	10.6	10.2	7.6	5.6	4.8	4.8
K10	52.7	-	57.3	56.9	11.6	-	10.2	10.4	64.3	-	67.5	67.3	11.6	-	10.1	9.6	4.5	-	5.6	5.5
K11	57.9	70.1	54.6	55.6	9.4	11.9	13.2	11.7	67.3	82.0	67.8	67.3	11.4	13.4	9.9	9.8	6.2	5.9	4.1	4.8
Mean[d]	64.1	67.9	60.1	60.9	10.5	10.6	9.1	11.1	74.5	78.5	69.1	72.0	12.4	13.3	10.4	10.1	6.5	6.8	7.1	5.8
CV (%)[e]	4.3	2.6	4.9	3.9	5.0	3.7	5.7	5.9	-	-	-	-	0.9	0.6	0.7	0.8	-	-	-	-

[a] Mean value (triplicate determinations)
[b] For abbreviations of cultivars see Table S1
[c] GLIA/GLUT, ratio between gliadins and glutenins
[d] mean value of each analytical parameter within one wheat species grown at one location
[e] Coefficient of variation across 15 cultivars of each wheat species grown at one location

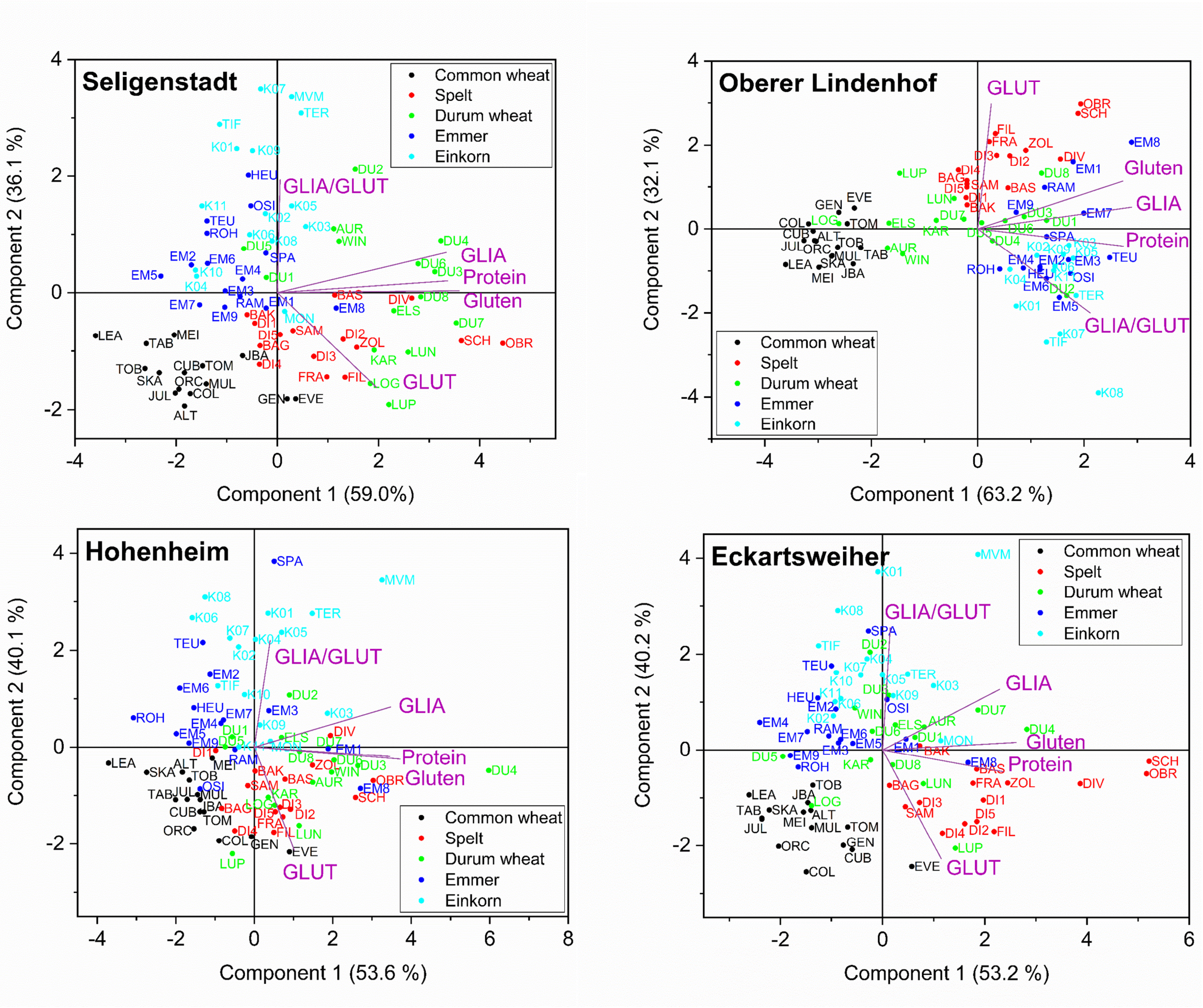

Figure S1. Biplot of principle component analysis (PCA) of gliadins (GLIA), glutenins (GLUT), gluten, protein content and ratio between GLIA and GLUT (GLIA/GLUT) of the five wheat species common wheat, spelt, durum wheat, emmer and einkorn for the four locations Seligenstadt, Oberer Lindenhof, Hohenheim and Eckartsweiher.

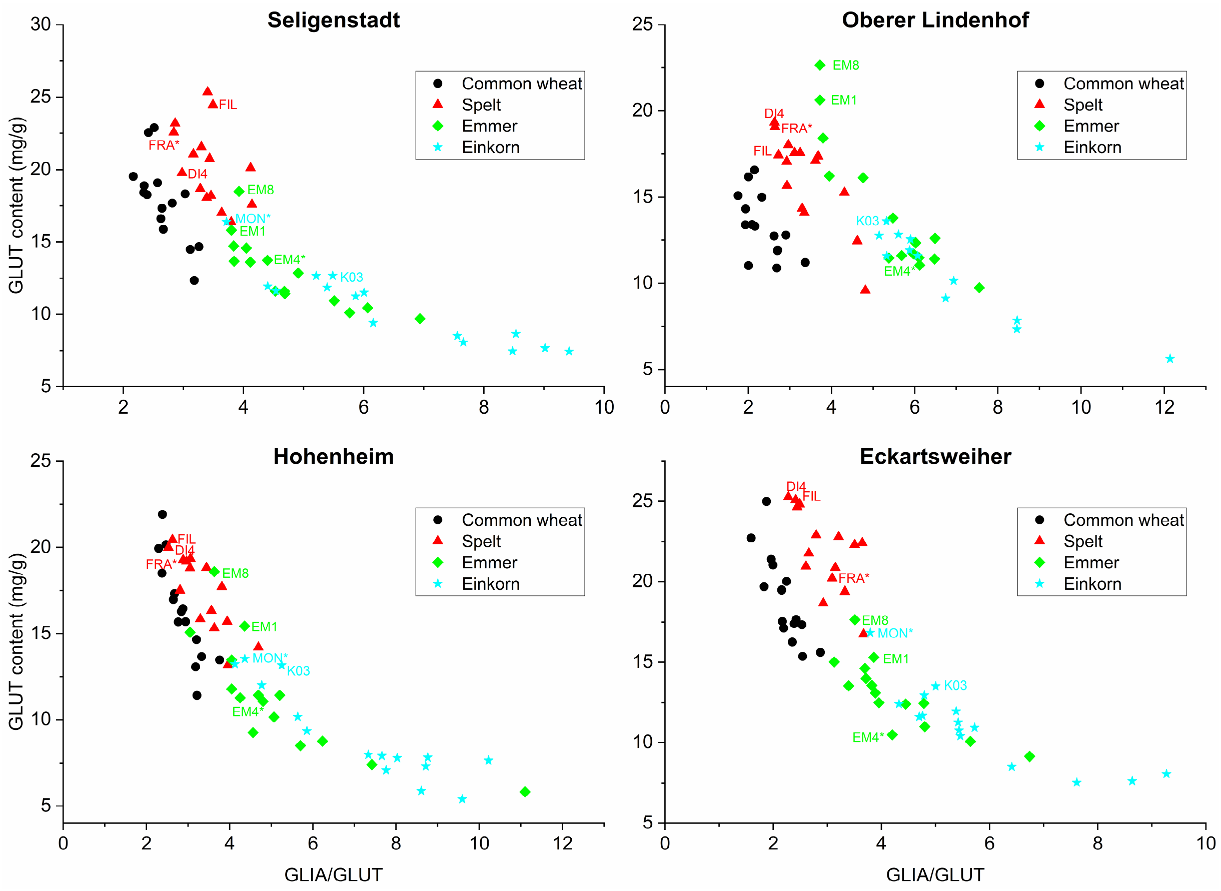

Figure S2. Scatterplot of glutenin content (GLUT) versus ratio of gliadins and glutenins (GLIA/GLUT) of common wheat, spelt, emmer and einkorn for the four locations Seligenstadt, Oberer Lindenhof, Hohenheim and Eckartsweiher for the identification of cultivars with predicted good baking performance.
